# Partial Splenectomy for a Sizeable Cavernous Hemangioma: Case Report and a Review of the Literature

**DOI:** 10.7759/cureus.12882

**Published:** 2021-01-24

**Authors:** Hatim Lazaar, Yosra Malki, Tariq Bouhout, Badr Serji, Tijani El Harroudi

**Affiliations:** 1 Surgical Oncology, Mohammed VI University Hospital, Regional Oncology Center, Oujda, MAR

**Keywords:** partial splenectomy, hereditary spherocytosis, overwhelming postsplenectomy sepsis, crush-clamp, spleen preservation, laparoscopic splenectomy, splenic cavernous hemangioma, overwhelming post-splenectomy infection, spleen preserving, total splenectomy

## Abstract

The recent awareness of the spleen's important role, especially its immune function, has fundamentally changed the management of splenic diseases, promoting the splenic preserving surgery, and protecting from the significant risk of total splenectomy: overwhelming post-splenectomy sepsis.

Partial splenectomy is a safe and feasible technique that offers, according to the literature, the same results of a total approach, either in achieving hematological benefits in congenital hemolytic anemia, or treating the focal splenic lesion such as hemangioma, while preserving the immune function.

## Introduction

The spleen is the largest secondary lymphoid organ; it has numerous immune response roles, including the clearance of affected or damaged cells from the bloodstream and host resistance to infection [1].

With this recent awareness of its importance and to prevent the overwhelming post-splenectomy infection, the therapeutic strategy of the splenic affections has fundamentally changed. It has led to preserve the spleen, either with the nonoperative management of the splenic trauma or the partial splenectomy.

Herein, we describe a case of cavernous splenic hemangioma successfully treated by an open partial splenectomy.

## Case presentation

**Figure 1:**
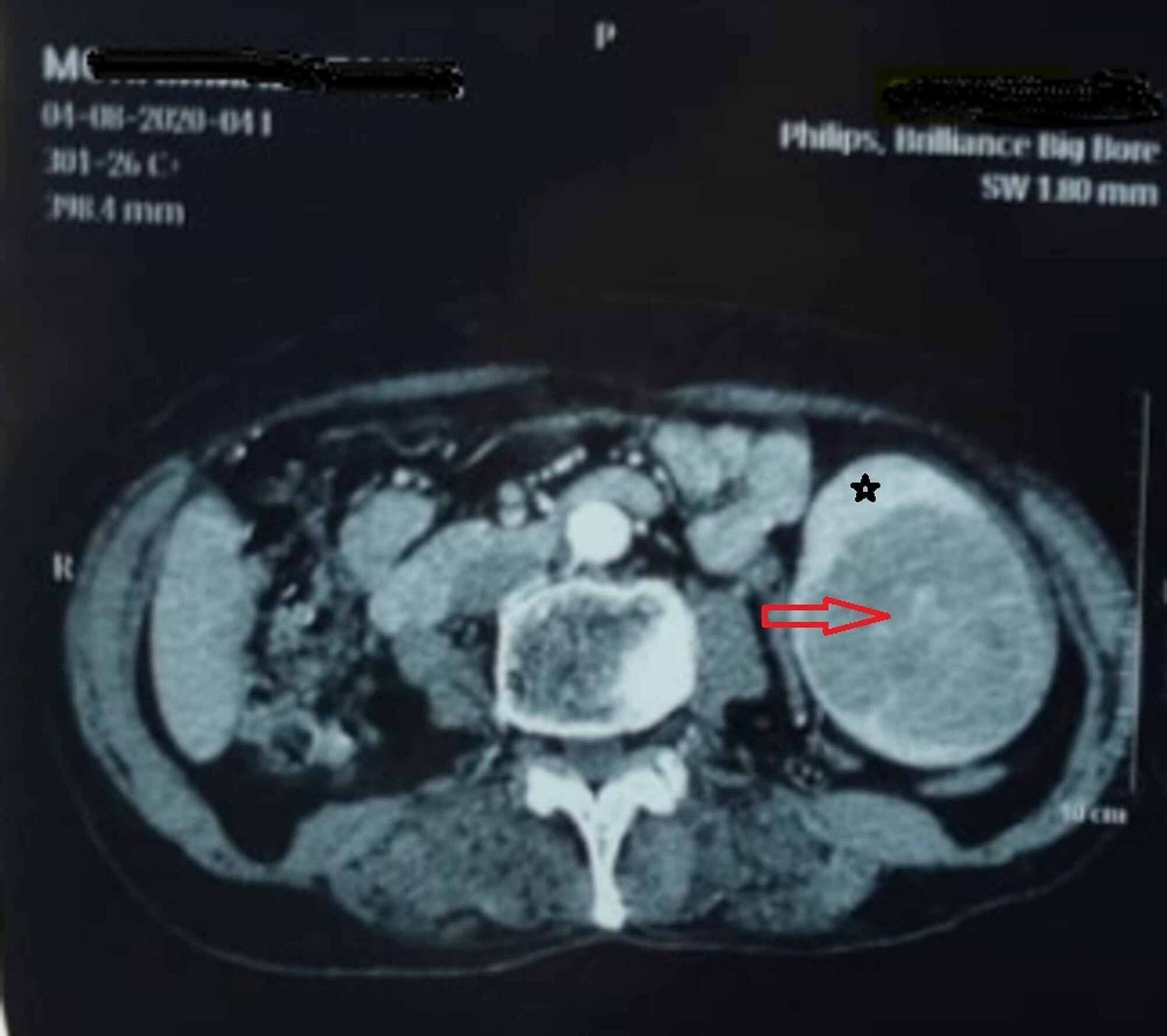
CT scan imaging revealing an inferior splenic hemangioma. Red arrow: Hemangioma Black star: Spleen

A 70-year-old female without a past medical history was admitted with chronic pain in the left upper quadrant, the physical examination was unremarkable, and laboratory findings were within normal limits. Unenhanced computed tomography (CT) scan showed a low-attenuation mass, enhanced CT image of the splenic lesion shows peripheral enhancement with a slow filling of contrast (Figure 1).

**Figure 2:**
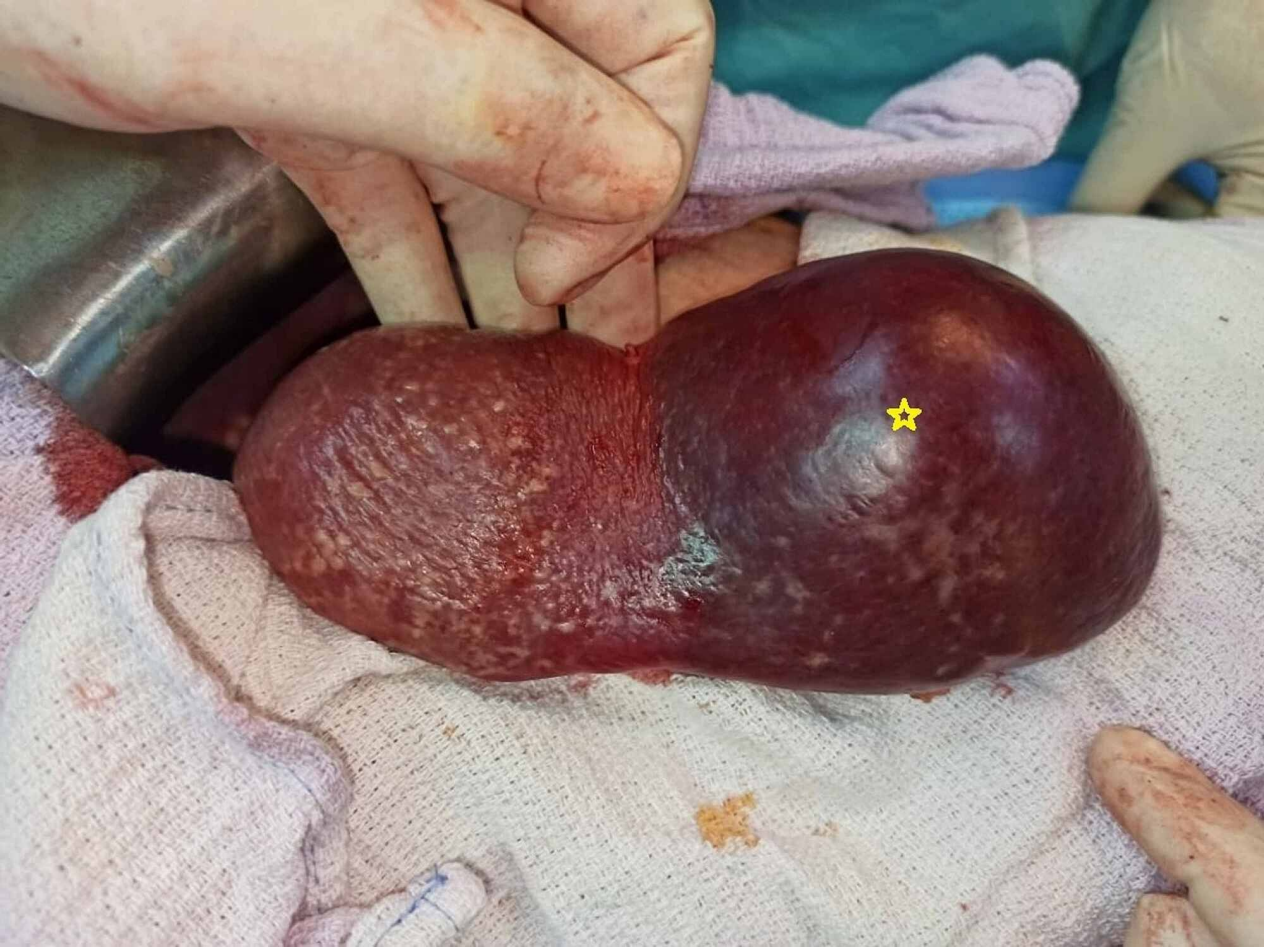
The ischemic area created after the ligation of the inferior splenic vessels (Yellow star: ischemic area).

**Figure 3:**
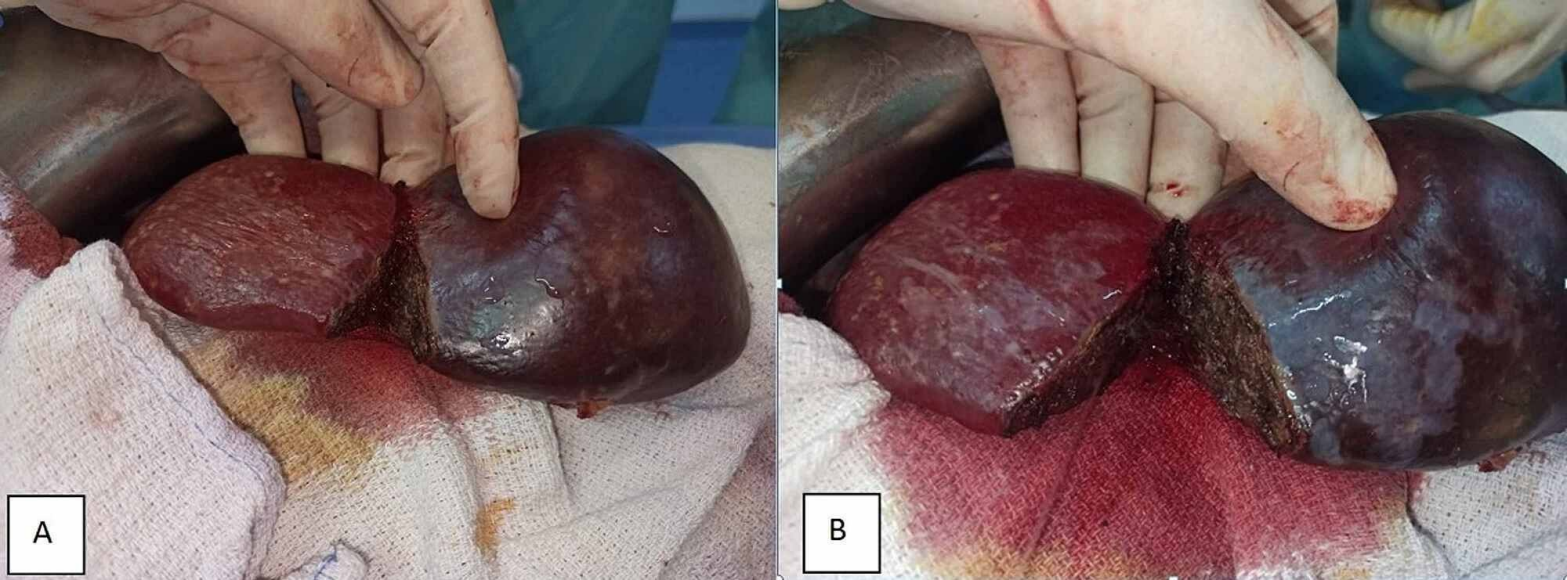
(A, B) Dissection of the splenic parenchyma using the crush-clamp technique.

The patient underwent open surgery, and the demarcation area was evident after segmental splenic vessel ligation (Figure 2). We started the dissection of the splenic parenchyma using the crush-clamp technique with Kelly to fracture the parenchyma and expose the vessels, along the demarcation line, with bipolar coagulation (Figure 3A, 3B).

The patient was discharged on the 5th day. We administered pneumococcal vaccination, Haemophilus influenzae type b conjugate vaccine, meningococcal conjugate vaccine, and Influenza immunization two weeks after surgery.

**Figure 4:**
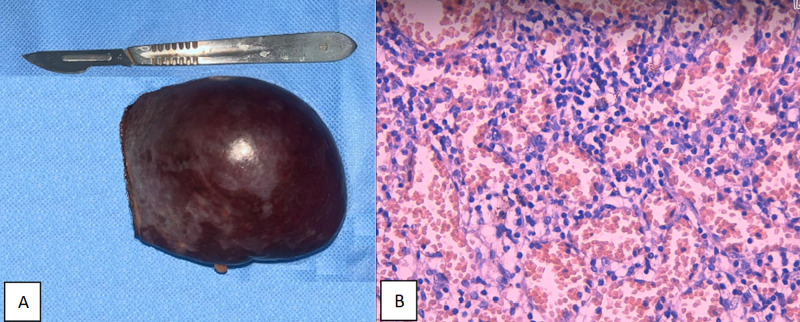
(A) Partial splenectomy with the resected splenic cavernous hemangioma. (B) Anatomopathological examination: Non-encapsulated non-neoplastic vascular channels, with vessels lined with a single layer endothelium.

The histopathological examination confirmed the splenic hemangioma showing a non-encapsulated non-neoplastic vascular channels, with vessels lined with a single layer endothelium (Figure 4A, 4B).

## Discussion

The spleen is the largest secondary lymphoid organ. Its anatomical, histological architecture and direct connection to the bloodstream confers a dual capacity to trap antigen and activate a rapid humoral immune response to provide the necessary opsonizing antibody for optimal bacterial clearance [1].

The discovery of the spleen's essential immunologic functions considerably changed the splenic surgery strategy; total splenectomy leaved room for splenic-preserving surgery to prevent overwhelming post-splenectomy sepsis and severe infections [2].

Splenic hemangioma (SH) is the second most common focal lesion involving the spleen after simple splenic cysts and the most prevalent benign primary neoplasm. It is described as a splenic slow flow venous malformations. Most SHs are discovered incidentally, especially in the second and third decade of life. The potential of malignant transformation to an angiosarcoma is unknown; therefore, treatment is required only for symptomatic or large ones.

Many therapeutic options are possible; splenectomy, either by laparotomy or laparoscopy, remains the best choice [3]. The first successful partial splenectomy via the open approach was reported in 1980 by Morgenstern and Shapiro. The same procedure was performed via laparoscopy in 1995 by Uranues [4,5].

Partial splenectomy is a therapeutic option gaining more and more place in the approach of focal splenic tumors, benign primary lesions such as cysts or hamartomas, iatrogenic injury to the spleen, metastases, and hematological diseases such as hereditary spherocytosis [6,7].

Besides, thanks to technological advances of instruments and laparoscopic surgery techniques, the laparoscopic approach has proven to be superior to the open approach, considering it a gold standard, mostly due to its advantages in postoperative pain control and decreased length of stay [4,5,8]. Moreover, as indicated in a systematic review of Costi et al., the number of laparoscopic approaches had considerably increased since 2006, translating into the notable rise in published papers [4].

Though, many studies report that laparoscopic partial splenectomy benefits may not be as apparent as total splenectomy, resulting in longer operative time, extended hospitalization, and more postoperative pain [4].

Recent papers also suggest that laparoscopic partial splenectomy is a safe and feasible technique for children with hematologic splenic diseases such as hereditary spherocytosis for achieving hematologic benefits [4,8,9].

In light of the incertitude of splenic function preservation achieved by the sparing techniques, preoperative preparation for a partial splenectomy should be the same as for a total splenectomy [10], consisting of a presurgical immunization with polyvalent pneumococcal, meningococcal, Haemophilus influenza type B, and influenza vaccines, which are recommended at least two weeks before surgery; if not administrated, they can be given one or two weeks later [10,11].

Additionally, life-long prophylactic antibiotics should be given to patients at high risk of pneumococcal infection. However, in low-risk patients, we should evaluate the benefit-risk balance of lifelong antibiotics, and patients may discontinue prophylaxis after a minimum of two years of treatment [11].

The surgical technique is based on the ligation of segmental vessels at the hilum, leading to a selective devascularisation of splenic parenchyma, as shown in Figure 2. This ischemic area is transected along the demarcation line [4], ensuring that at least 25% of the spleen is left with sufficient perfusion to preserve its immune function [6].

According to a recent metanalysis, the conversion risk from laparoscopy to laparotomy is around 6.4%, while the conversion from partial to total splenectomy is 3.4% [4].

Most papers published confirm that partial splenectomy may be the new alternative to total splenectomy whenever possible. However, this topic's major drawback is the inherent limitations of retrospective studies, the small sample sizes, and the lack of consensus.

## Conclusions

Partial splenectomy is a splenic preserving surgery gaining more place in the last two decades, offering the same hematologic and therapeutic results, with fewer risks of overwhelming post-splenectomy infection.
